# Subject and Object Pronouns in High-Functioning Children With ASD of a Null-Subject Language

**DOI:** 10.3389/fpsyg.2019.01301

**Published:** 2019-06-04

**Authors:** Arhonto Terzi, Theodoros Marinis, Anthi Zafeiri, Konstantinos Francis

**Affiliations:** ^1^Technological Educational Institute of Western Greece, Patras, Greece; ^2^Department of Linguistics, Universität Konstanz, Konstanz, Germany; ^3^School of Psychology and Clinical Language Sciences, University of Reading, Reading, United Kingdom; ^4^Kuwait Centre for Mental Health, Shuwaikh, Kuwait

**Keywords:** autism, subject pronouns, object pronouns, null subject languages, Greek

## Abstract

Although the use of pronouns has been extensively investigated in children with autism spectrum disorders (ASD), most studies have focused on English, and no study to date has investigated the use of subject pronouns in null subject languages. The present study aims to fill this gap by investigating the use of subject and object pronouns in 5- to 8-year-old Greek-speaking high-functioning children with ASD compared to individually matched typically developing age and language controls. The “Frog where are you” (Mayer, 1969) narrative task was used to elicit subject and object pronouns as well as Determiner Phrases (DPs). Greek is a null subject language, and as a result, subject pronouns most often remain without phonological content. The findings showed that both groups used more null than overt subject pronouns, indicating that children with ASD know that Greek is a null subject language. TD children used more null subjects than subject DPs, whereas children with ASD used an equal proportion of null subjects and subject DPs. In terms of object pronouns, both groups produced more clitics and object DPs than strong object pronouns, but the difference between clitics and DPs did not reach significance in either of the groups. Importantly, the groups did not differ from each other in the use of ambiguous pronouns in both the subject and object position. The ASD children’s avoidance to use pronominal subjects can be taken as evidence that they use a strategy to avoid infelicitous reference. This would suggest that the ASD children’s difficulties with pronouns is not due to difficulties in core grammar.

## Introduction

The use of pronouns is a domain that has triggered a considerable amount of research in the context of individuals with autism spectrum disorders (ASD). We suspect this is so because it is a process that implicates domains of grammar at which individuals with ASD usually fall behind (see e.g., Marinis et al., 2013). For instance, pronoun reference requires making pragmatic judgments about what is prominent in the discourse, given that a pronoun picks up as its antecedent a prominent noun phrase in the immediately preceding discourse, and it takes into account the perspective of the listener (see Hendriks et al., 2014 for much related discussion).

The literature in ASD has so far focused almost exclusively on the reference of subject pronouns and has found that even individuals with high-functioning autism and verbal abilities within the normal range fall behind their typical controls in two ways: either they produce more infelicitous pronouns than typical controls, in the sense of pronouns with no clear reference to some antecedent in the discourse, or they produce proportionally more Determiner Phrases (DPs) than the corresponding pronouns. For example, the studies of Norbury and Bishop (2009), Novogrodsky (2013), and Novogrodsky and Edelson (2016) resulted in findings of the former type (use of infelicitous pronouns) by investigating the behavior of 6;0–10;0 (mean age:8;8) and 6;1–14;3 (mean age:10) year-old individuals with ASD, while the studies of Colle et al. (2008) and Arnold et al. (2009) have reached conclusions of the latter type (more full DPs than pronouns) by studying the behavior of adults and 11–15 year-old adolescents, respectively.

While the participants of the above studies have different characteristics, at least in terms of their age, the studies share two important properties: (a) the language investigated by all of them is English and (b) with the exception of Novogrodsky and Edelson (2016), the rest of the studies investigate the reference of subject pronouns. Novogrodsky and Edelson (2016) investigated English object pronouns as well, along with subject and possessive pronouns. Object pronouns, in particular, the clitic object pronouns of Greek, were also investigated by Terzi et al. (2017). Both studies did not find important differences on object pronouns between children with ASD and their typical controls, but they attribute the similar performance of the two groups to different reasons. Novogrodsky and Edelson (2016) suspect that the finding may be a consequence of the small body of the object pronoun data that constituted their sample, while Terzi et al. (2017) attribute the similar performance of children with ASD and their typical controls to the fact that the TD children were too young to have fully mastered the referential abilities of these pronouns, as supported by the fact that they had several infelicitous cases of pronouns. Note that the high-functioning children with ASD and their well-matched typical controls were aged 5;11–8;8 (*M* = 6;11) in the latter study, while it has been claimed that young children acquire the use of pronouns with clear antecedents during elementary school and certainly not before the age of 7 (Hendriks et al., 2014), or even before the age of 9 according to Berman (2009).

In contrast to the acquisition of object pronouns by Greek-speaking children with ASD, not much is known about the acquisition of subject pronouns. It is, therefore, unclear whether felicitous reference of pronouns varies according to their position in the sentence in Greek as well, much like in the study of Novogrodsky and Edelson (2016) for English. Such an investigation is important not only for the sake of comparing the two fundamental positions of the sentence in Greek, but also because Greek, by contrast to English, (1a), is a null-subject language, hence, subject pronouns most often remain without phonological content (1b).

(1) (a) ^∗^(She) loves Kostas.
(b) agapa ton Kosta
  love.3s the.acc Kostas.acc
  “ (She) loves Kostas.”

Overt pronouns in the subject position are very rare in adult typical Greek, and there is no evidence that things are different in child language^1^. It is difficult to say whether this characteristic, which is certainly not unique to Greek, is expected to make things easier or more difficult for children with ASD. But the distinction into null and non-null-subject languages is a fundamental one in linguistic theory, and it is one that has not been investigated before in the language of individuals with ASD (who are known to have difficulties with pronouns in various ways). It is, therefore, worth undertaking the task of investigating the use of subject pronouns in Greek, in order to see whether children with ASD use null and overt subject pronouns in a similar manner or proportions as TD populations, in addition to investigating how the pattern of Greek compares to that in the study by Novogrodsky and Edelson (2016) for English with respect to how subject vs. object pronouns pick their reference, and how many pronouns overall are used in relation to nouns (i.e., DPs).

To elaborate a bit more on the findings of Terzi et al. (2017), the authors focused on the reference of object pronouns, which in Greek employ a shorter form, namely, a pronominal object clitic as *ton* “him” in (2b), instead of the corresponding DP, or strong pronoun, (2a) (see also footnote 1).

(2) (a) I Maria agapa ton Kosta/afton
  the.nom Mary.nom love.3s the.acc Kostas.acc/him
  “Mary loves Kostas/him.”
(b) I Maria *ton* agapa.
  the.nom Mary.nom him.clitic love.3s
  “Mary loves him.”^2^

The purpose of the Terzi et al. (2017) study was to compare the behavior of children with ASD in a narrative task, which elicits language samples that are very close to spontaneous language, to their behavior on a structured task they had undertaken with the same children, which elicited the production of object clitic pronouns (Terzi et al., 2016a,b). The production task revealed that the children with ASD fell behind their individually language matched TD controls on the production of object clitic pronouns and on selecting the target referent for them, but although the difference between the two groups was statistically significant, it was small (Terzi et al., 2016a,b). On the other hand, Terzi et al. (2017) found that the same children with ASD did not fall behind their matched TD controls in their use of object clitic pronouns in a narrative task, in terms of associating object clitics with a felicitous antecedent. The only difference between the two groups was that the children with ASD tended to select as the referent of object clitic pronouns items that occupied the subject position of the previous sentence, by contrast to the object clitic pronouns of the TD children whose referents were either in the subject or the object position in the previous sentence. Terzi et al. (2017) attributed the finding that the two groups essentially differed only in the structured tasks to the fact that, for each item to which they responded, they had to take the experimenter’s perspective and input into consideration because the discourse representation was not the same across items and conditions. In contrast, although in the narrative task participants also had to take the experimenter’s point of view into consideration, the discourse representation did not change during the task and participants had full control of the discourse. This made the structured tasks more challenging than the narrative because children had to be constantly alert for a potential change in the discourse representation from each item to the next.

The purpose of this study is to extend the research on the acquisition of pronouns by Greek-speaking children with ASD to subject pronouns. In particular, first, it aims to investigate whether the null vs. overt subject pronouns ratio of children with ASD differs from that of TD children, which amounts to asking whether children with ASD know that Greek is a null-subject language. Secondly, it aims to address whether children with ASD differ from their TD controls in terms of whether their subject pronouns (either null or overt) select felicitous referents. Finally, it aims to uncover whether the pattern attested in subject pronouns in some of the English studies in terms of ratio of pronouns vs. DPs is also present in Greek, either for subject or for object pronouns and whether this ratio differs between the children with ASD and the typically developing children.

## Materials and Methods

### Participants

The participants of this study are the same as those in Terzi et al. (2016a,b, 2017). The children are 20 high-functioning Greek-speaking children with ASD, aged 65–104 months (mean age: 6;11), matched on their verbal abilities on the basis of the Greek Peabody Picture Vocabulary Test (PPVT) (Simos et al., 2011) with 20 typically developing (TD) children, aged: 61–98 months (mean age: 6;7). The first group of children were attending private clinics in Athens and Patras specialized in children with ASD, and were holding a community diagnosis of a Pervasive Developmental Disorder (PDD) according to DSMIV-TR criteria (American Psychiatric Association, 2000). The child psychiatrist of our team (KF), an ADOS trainer, corroborated the diagnosis with the use of Autism Diagnostic Observation Schedule, Second Edition - ADOS-2 (Lord et al., 2012). Ethical approval for the study was provided by the Research Ethics Committee of the Ministry of Education (Institute of Educational Policy). All parents provided informed written consent for their children’s participation.

The children of both groups were also administered a number of baseline tasks: the Raven’s Colored Matrices test (Raven et al., 2008), assessing their non-verbal abilities, the production of morphosyntax subtest of the Diagnostic Test of Verbal Intelligence (DVIQ) (Stavrakaki and Tsimpli, 2000), and two tasks that assessed phonological and working memory (forward and backward digit span)^3^. Table 1 shows the scores of the two groups on the verbal and non-verbal tasks, and see Terzi et al. (2016b) for their memory scores, on which the two groups did not differ from each other.

**Table 1:** Baseline tasks.

		Children with ASD	TD children	*p*-value
Raven’s standard score	Mean	104.8	95.5	<0.05
	Range	80–130	80–115	
	SD	18.2	7.9	
PPVT raw score	Mean	92.9	93.1	>0.1
	Range	76–123	74–122	
	SD	14.9	14.7	
DVIQ raw score	Mean	20.8	21.4	>0.1
	Range	15–24	17–24	
	SD	2.3	2.1	

There was no significant difference between the two groups on their vocabulary abilities based on the PPVT [*F*(1, 39) = 0.003, *p* = 0.958, η^2^ < 0.001] and on their grammatical abilities based the DVIQ [*F*(1, 39) = 0.87, *p* = 0.357, η^2^ = 0.022]. The two groups differed significantly from each other only on their non-verbal IQ (Raven et al., 2008), on which the ASD children scored slightly better than their TD controls [*F*(1, 39) = 4.324, *p* = 0.044, η^2^ = 0.102]. This is why the score of the Raven’s was used as a co-varying factor in the analyses of the narrative task.

### Materials

The two groups of children had to narrate the story “*Frog where are you*” from the illustrated book of Mayer (1969). This is a book with pictures only, telling the story of a boy, a dog, and a pet frog. One night the frog leaves his jar and the boy with his dog undertake a whole adventure in order to find him. We used this book because it has been used in a number of related studies (Colle et al., 2008; Norbury and Bishop, 2009), as well as in the study of Novogrodsky and Edelson (2016), which is more closely related to ours. The person who used the book with the children was the same person that administered the structured experiments. She gave each child the book and told them that it was a book about a child, his dog, and his frog and that they would look at it together first, by just turning the pages. Then, the children were asked to look at the book from the beginning again and tell the experimenter what was going on in each page. The experimenter was not looking at the book during this stage, but was making sure the narratives were being audio-recorded.

Two coders undertook the transcription and annotation of the narratives for the purposes of their thesis (Grammatikou and Karamali, 2015). The third author of this article read all transcriptions against the recordings for the purposes of her thesis and verified accuracy with an interrater reliability of 95% (Zafeiri, 2016). Referring expressions in each narrative were coded for their grammatical function (subject, object, other), while subjects were coded as null, overt, or DPs, and objects as strong pronouns, clitics, or DPs. Ambiguous referents were defined as referents for which either no referent or more than one referents were available. The transcripts were transferred into CHAT format by Konstantina Olioumtsevits as part of her Erasmus placement at the University of Reading. The second author checked the CHAT format and calculated the children’s MLU. The children with ASD had similar MLU (Mean = 8.52) to the TD children (Mean = 8.83) [*F*(1, 40) = 1.472, *p* = 0.233, η^2^ = 0.038].

The data were analyzed using repeated measures ANOVA with Group as a between subjects factor, Type as a within subjects factor, and the score of the Raven’s as a co-variable because of the difference between the two groups on the Raven’s score (see Table 1).

## Results

**FIGURE 1:**
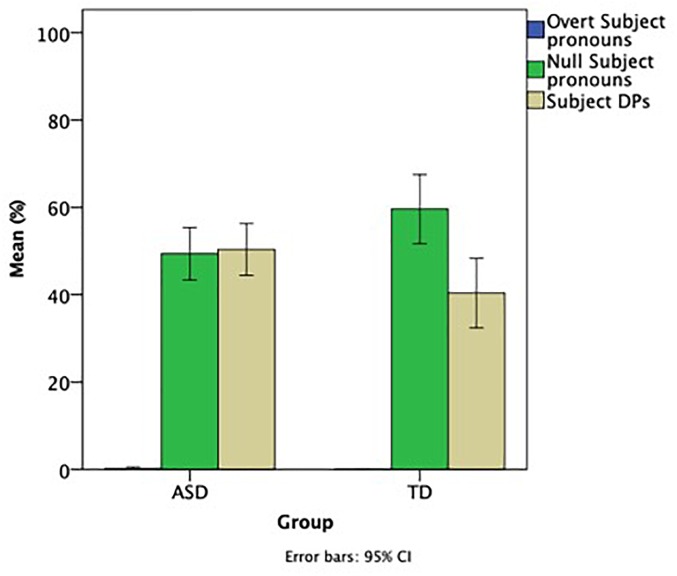
Ratio of null, overt subject pronouns and subject DPs.

Figure 1 provides an overview of the use of null and overt subject pronouns, as well as subject DPs, in the groups of children with ASD and their TD controls, and addresses the first aim, i.e., whether children with ASD know that Greek is a null-subject language. The actual numbers of overt pronouns, null pronouns, and subject DPs for the two groups was as follows. Overt pronouns: ASD = 5, TD = 10; Null pronouns: ASD = 431, TD = 481; Subject DPs: ASD = 421, TD = 354.

A quick look at Figure 1 shows that the vast majority of subjects in both groups were null pronouns and DPs, whereas an extremely small number of subjects consisted of overt pronouns. To address differences between the two groups using inferential statistics, a mixed ANOVA with group (ASD, TD) as a between group factor, subject type (overt, null, DP) as a within group factor, and Raven’s as a co-variable showed no significant main effect of Type, Group, or Raven’s, no significant interaction between Type and Raven’s, but there was a significant interaction between Group and Type [*F*(2, 74) = 3.849, *p* = 0.026, η^2^ = 0.094], demonstrating that the two groups showed a different pattern of performance.

In particular, both groups used massively more null than overt subject pronouns, indicating that children with ASD know that Greek is a null-subject language. In children with ASD, there was a significant difference between overt pronouns and null pronouns (*p* < 0.001), as well as subject DPs (*p* < 0.001), but no significant difference between null pronouns and subject DPs (*p* = 1). In contrast, TD children also showed a significant difference between overt pronouns and null pronouns (*p* < 0.001), as well as subject DPs (*p* < 0.001), but the difference between null pronouns and subject DPs was approaching significance (*p* = 0.061), a consequence of the fact that the TD children used more null subjects than subject DPs. A comparison between the two groups in each type of subjects with Raven’s as a co-varying factor showed that there was no effect of Raven’s in null subjects, overt subjects, or subject DPs. TD children used more null subjects than children with ASD [*F*(1, 39) = 3.949, *p* = 0.054, η^2^ = 0.096] and the opposite pattern was approaching significance for subject DPs [*F*(1, 39) = 3.749, *p* = 0.061, η^2^ = 0.092], i.e., children with ASD used more subject DPs than TD children. The two groups did not differ in the use of overt pronouns.

**FIGURE 2:**
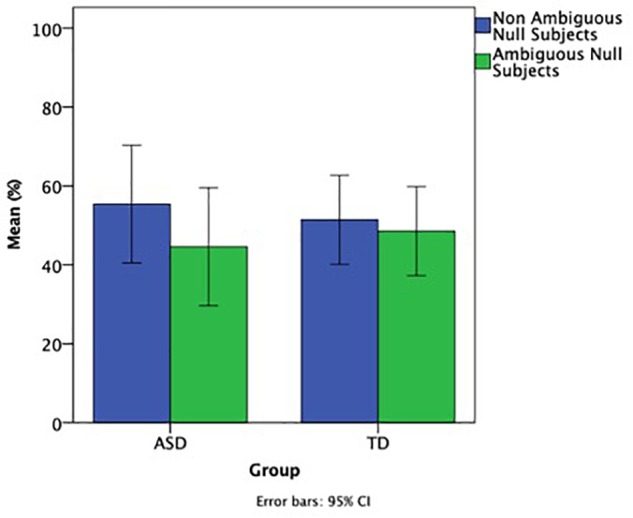
Ratio of ambiguous and non-ambiguous null subject pronouns.

To address the other aim of this study, i.e., whether children with ASD differ from their TD controls in terms of their subject pronouns selecting felicitous referents, we compared felicitous vs. non-felicitous use of subject pronouns in the two groups. Given the very low number of overt subject pronouns in both groups (5 out of 436 for children with ASD and 10 out of 491 for TD children), we focused on null pronouns only and compared the percentage of their ambiguous and non-ambiguous instantiations, shown in Figure 2. The exact numbers for ambiguous and non-ambiguous null subjects follows. Ambiguous null subjects: ASD = 156, TD = 235; unambiguous subjects: ASD = 275, TD = 246.

Representative examples of infelicitous pronominal reference of children from the ASD group are shown below.

(3) Tin ora pu kimotan to pedhaki, o vatrahos efighe the time that was-sleeping the little-child, the frog left apo to parathiro from the window.
(4) (a) Meta epsakse padu. then *pro* searched.3s everywhere. *pro* Epsaksan padu. searched.3p everywhere.
(b) Molis tus *pro* ide, *pro* espase to vazo
  when *them*.clitic saw.3s, broke.3s the vase
  “When he/she saw them, he/she broke the vase.” (Child: 101)

In the above extract from the child’s narrative, the referent of the 3rd singular null subject pronouns (*pro*) in (4a) is not clear, as it could conceivably be either the child or the frog cf. (3). On the other hand, there is no obvious referent for the 3rd plural null pronoun in (4b). The referent of the plural null pronoun is most probably *the child and his dog*, because we know from the story that they both looked for the frog; however, *the dog* has not been mentioned in this part of the narrative. Notice, that there is also an instance of object clitic pronoun with infelicitous reference in (4b), *tus* “them”, a plural object pronoun but no possible plural referent. An extract from another child’s narrative follows below:

(5) (a) Experimenter: ti vlepis?
  what see.2s
  “What can you see?”
(b) Child: ena skilaki, ena vatracho ke ena pedhaki.
  a little-dog, a frog and a little-child.
  “A dog, a frog and a child.”
(6) otan *pro* kimotan, *ton* pighe o skilos when was-sleeping.3s, him.clitic took.3s the dog piso tu ke *ton pro* ksipnise. behind his and him.clitic woke up-3s
  “When (he/she) was asleep, the dog followed him and woke him up.”
  (Child: 119)

In (6) there are two instances of the pronominal object clitic *ton* “him,” but two potential referents in the preceding context, i.e., *the frog* and *the child*. The referent of the null subject of *kimotan* “was asleep” is also not clear, further complicating the reference of the pronominal clitics. For more such examples, as well as examples of pronominal object clitics with felicitous reference see Terzi et al. (2017).

A mixed ANOVA with group (ASD, TD) as a between group factor, pronoun type (ambiguous, non-ambiguous) as a within group factor, and Raven’s as a co-varying factor showed no significant difference between the two groups, no significant difference between ambiguous and non-ambiguous pronouns, no significant effect of Raven’s and no interactions between these factors, demonstrating that the two groups showed a similar pattern of behavior.

**FIGURE 3:**
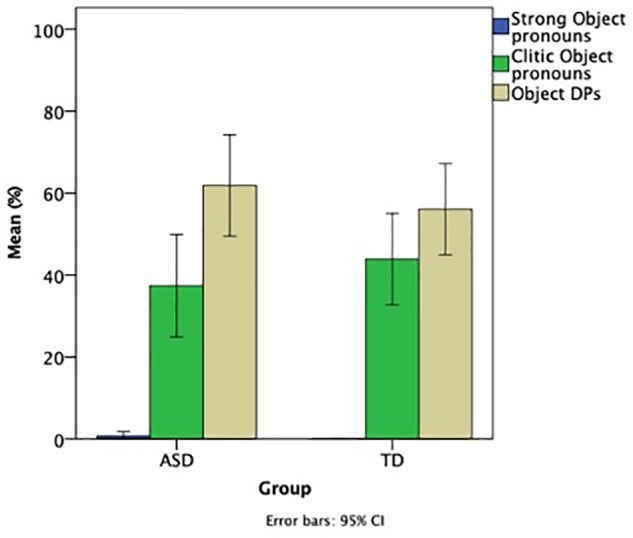
Ratio of strong and clitic object pronouns as well as object DPs.

To address the final aim, namely, whether there is a difference between subject and object (clitic) pronouns in terms of selecting a felicitous referent, and in terms of whether the two groups differ in the number of pronominal objects and DP objects they use, we analyzed the narrative data for objects in the same manner as for subjects. Figure 3 shows the strong object pronouns, clitic object pronouns, and object DPs. The exact numbers of strong object pronouns, clitic object pronouns, and object DPs for the two groups were as follows. Strong pronouns: ASD = 2, TD = 0; Clitic pronouns: ASD = 88, TD = 115; Object DPs: ASD = 153, TD = 142.

As with subjects before, very few overt strong pronouns were used in the object position. The vast majority of object referring expressions in both groups were clitic pronouns and object DPs. To address the differences between the two groups using inferential statistics, a mixed ANOVA with group (ASD, TD) as a between group factor, object type (clitic, strong, DP) as a within group factor, and Raven’s as a co-varying factor showed only a significant main effect of Type [*F*(2, 36) = 144.575, *p* < 0.001, η^2^ = 0.889], but no significant effect Group, or Raven’s, and no significant interactions between the factors. The main effect of type was attested because both groups of children produced more clitics and DPs vs. strong pronouns (both comparisons *p* < 0.001) but the difference between clitics and DPs did not reach significance (*p* = 0.083) in either group. This demonstrates that the two groups showed a similar pattern of performance in terms of the numbers of object pronouns and object DPs they used.

**FIGURE 4:**
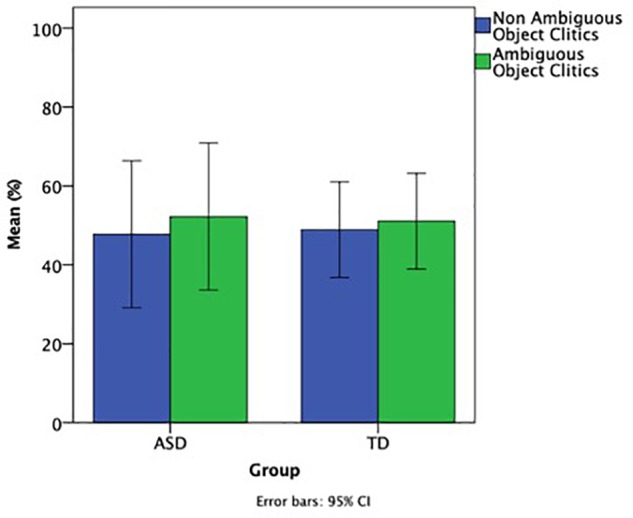
Ratio of ambiguous and non-ambiguous object clitic pronouns.

As with subject pronouns before, in order to address if the children with ASD differ from their TD controls in terms of whether object pronouns select felicitous referents, we focused only on clitic pronouns because of the very low number of strong object pronouns in both groups (2 out of 90 for children with ASD and 0 out of 115 for TD children), and compared the percentage of ambiguous and non-ambiguous object clitic pronouns, shown in Figure 4. The exact numbers for ambiguous and non-ambiguous clitic pronouns for the two groups was ambiguous clitics: ASD = 47, TD = 62; unambiguous clitics: ASD = 41, TD = 53.

A mixed ANOVA with group (ASD, TD) as a between group factor, pronoun type (ambiguous, non-ambiguous) as a within group factor, and Raven’s as a co-varying factor showed no significant difference between the two groups, no significant difference between ambiguous and non-ambiguous pronouns, no significant effect of Raven’s and no interactions between these factors, demonstrating that the two groups showed a similar pattern of performance in terms of felicitous reference of the object clitics.

## Discussion

This study addressed the use of subject and object pronouns in narratives, as they are employed by Greek-speaking children with ASD and their TD controls. To our knowledge, no study other than that of Novogrodsky and Edelson (2016) has investigated the reference of pronouns in discourse, both in the subject and the object position, neither the actual numbers of pronouns as opposed to full DPs. Moreover, no study has investigated the use of null or overt subject pronouns of children with ASD who grow up speaking a null-subject language.

The first aim of the study was to investigate whether the null vs. overt subject pronouns ratio of children with ASD differs from that of TD children, a difference which tells us whether children with ASD know that Greek is a null-subject language. Our study demonstrated that the children with ASD know this fundamental property of Greek, as it emerged from the very similar ratio of null vs. overt subject pronouns of the two groups, or else, the very low number of overt pronouns in both groups. This suggests that high-functioning children with ASD have no problem with core grammar, i.e., principles of the initial state of language faculty (and presumably, along with them, the knowledge that that there are also parameters that need to be set)^4^. This conclusion is in line with earlier claims of ours (Terzi et al., 2016a,b) that high-functioning children with ASD do not have problems with syntax proper, but with the interface of syntax with pragmatics (and possibly phonology).

The second aim of the study was to address whether children with ASD differ from their TD controls in terms of whether their subject pronouns select felicitous referents. Moreover, it aimed at uncovering whether there is a difference between subject or object pronouns in terms of selecting a felicitous referent, and whether this is different for the two groups. The data revealed that the proportion of ambiguous versus non-ambiguous pronouns does not differ between the two groups, regardless of whether they occupy a subject or an object position. We know this already for object pronouns (clitics) from the study of Terzi et al. (2017), but it was confirmed by the analysis conducted for the purposes of this article. Notice that the number of object pronouns is remarkably lower (children with ASD: *N* = 90, TD children: *N* = 115) than that of subject pronouns (children with ASD: *N* = 436, TD children: *N* = 491), yet the behavior of children with ASD does not differ depending on the position of the pronoun (subject or object). In other words, absolute number of pronouns was not able to affect the ratio of ambiguous vs. non-ambiguous pronouns of Greek-speaking children with ASD, contrary to an interpretation that had been given for English object pronouns by Novogrodsky and Edelson (2016), according to which the good performance of English-speaking children with ASD on them was due to their low numbers. The authors attributed the distinct behavior in position of pronouns and felicitous reference to the fact that their corpus contained many more pronouns in the subject than in the object position.

Finally, the ratio of pronouns vs. their corresponding DPs in the subject and object position was calculated for both groups, as this is another aspect in which the pronouns of individuals with ASD have been found to differ from those of typical populations in the literature. The main finding was that, by contrast to object pronouns, where both groups used the same proportion of pronouns vs. DPs, different proportions were used for subject pronouns and subject DPs by the two groups: whereas TD children used more pronouns than DPs (481/354), children with ASD used almost equal numbers (431/421) of null pronouns and DPs. Hence, the two groups do not differ in terms of selecting a felicitous referent for pronouns, but in terms of using more DPs rather their corresponding pronouns, although in the subject position only. Nevertheless, children with ASD did not differ in terms of using more overt than null pronouns in the subject position when compared to their controls, as already mentioned. If the latter is a matter of setting a UG parameter, while the former deciding which referent in the discourse is more prominent, and hence, which one can be replaced by a pronoun, the results suggest that the problem with pronouns in ASD is related to discourse and not to core grammar. Finally, given that the group of children with ASD in the present study were within the high-functioning range of the spectrum, only future research can address what pattern holds for low-functioning children or for language impaired children with ASD, because, with the exception of Colle et al. (2008), no other study has focused on such populations so far when it comes to pronominal reference.

## Conclusion

This study investigated the use of subject and object pronouns by Greek-speaking high-functioning children with ASD and compared it to the use of pronouns by TD age and language controls in a narrative task. The groups did not differ from each other in the use of ambiguous pronouns in both the subject and object position. The only difference between the two groups concerned the use of subject pronouns vs. subject DPs. Whereas TD children used more subject pronouns than subject DPs, the children with ASD used an equal proportion. The ASD children’s avoidance to use pronominal subjects can be taken as evidence that they use a strategy to avoid infelicitous reference, either because they cannot decide what the prominent element is in the discourse, or because they cannot see that there is a prominent element. In relation to the finding that children with ASD know when to use a null or an overt pronoun, and largely avoid the latter, we conclude that the ASD children’s difficulties with pronouns are not difficulties due to core grammar.

## Ethics Statement

This study was carried out in accordance with the recommendations of the Research Ethics Committee of the Greek Ministry of Education (Institute of Educational Policy) with written informed consent from the parents/guardians of all participants. The parents/guardians of all participants gave written informed consent in accordance with the Declaration of Helsinki. The protocol was approved by the Research Ethics Committee of the Greek Ministry of Education (Institute of Educational Policy). All parents provided informed written consent for their children’s participation.

## Author Contributions

AT, TM, and KF conceived and designed the study. AZ transcribed and coded the data. AT oversaw the implementation of the study and the data collection. TM conducted the statistical analyses of the data. AT and TM wrote the manuscript.

## Conflict of Interest Statement

The authors declare that the research was conducted in the absence of any commercial or financial relationships that could be construed as a potential conflict of interest.

## Footnotes

1 We would not want to say that overt subject pronouns are not an option in Greek, because they can be possible under specific pragmatic conditions (e.g., emphatic/contrastive). The same holds for strong object pronouns. A property of Greek strong pronouns is that they are homophonous to demonstratives, which is most probably the reason for the lack of delay of Principle B for strong pronouns in the object position, as demonstrated and discussed by Varlokosta (2000, 2008), respectively. This is by contrast to Spanish, for instance, where only object clitics, but not the corresponding strong object pronouns, avoid the delay of Principle B (Padilla, 1990, and work afterward).

2 For the distinction in strong pronouns, weak pronouns and clitics see Cardinaletti and Starke (1999).

3 The DVIQ consists of five subtests, assessing: expressive vocabulary, comprehension of morphosyntax, production of morphosyntax, sentence repetition, comprehension of metalinguistic concepts. We used the production of morphosyntax subtest, which consists of 24 items assessing number, gender, case, tense morphology, subject-verb agreement, prepositions, among a few other things, via sentence completion.

4 For more on “core grammar”, see Crain (2010), but see Hendriks et al. (2014) for a different view. See also di Sciullo and Agüero-Batista (2008) for a similar view to ours with respect to the delay of Principle B in typical language, i.e., that it is not a problem with UG/initial state of language faculty, or else, it is not a problem with Principle B per se.
